# RNA Editing and Drug Discovery for Cancer Therapy

**DOI:** 10.1155/2013/804505

**Published:** 2013-04-24

**Authors:** Wei-Hsuan Huang, Chao-Neng Tseng, Jen-Yang Tang, Cheng-Hong Yang, Shih-Shin Liang, Hsueh-Wei Chang

**Affiliations:** ^1^Department of Pharmacy, Kaohsiung Medical University Hospital, Kaohsiung Medical University, Kaohsiung 807, Taiwan; ^2^Graduate Institute of Natural Products, College of Pharmacy, Kaohsiung Medical University, Kaohsiung 807, Taiwan; ^3^Department of Biomedical Science and Environmental Biology, Kaohsiung Medical University, Kaohsiung 807, Taiwan; ^4^Department of Radiation Oncology, Faculty of Medicine, College of Medicine, Kaohsiung Medical University, Kaohsiung 807, Taiwan; ^5^Department of Radiation Oncology, Kaohsiung Medical University Hospital, Kaohsiung 807, Taiwan; ^6^Department of Electronic Engineering, National Kaohsiung University of Applied Sciences, Kaohsiung 807, Taiwan; ^7^Department of Biotechnology, Kaohsiung Medical University, Kaohsiung 807, Taiwan; ^8^Center for Resources, Research and Development, Kaohsiung Medical University, Kaohsiung 807, Taiwan; ^9^Cancer Center, Kaohsiung Medical University Hospital, Kaohsiung Medical University, Kaohsiung 807, Taiwan

## Abstract

RNA editing is vital to provide the RNA and protein complexity to regulate the gene expression. Correct RNA editing maintains the cell function and organism development. Imbalance of the RNA editing machinery may lead to diseases and cancers. Recently, RNA editing has been recognized as a target for drug discovery although few studies targeting RNA editing for disease and cancer therapy were reported in the field of natural products. Therefore, RNA editing may be a potential target for therapeutic natural products. In this review, we provide a literature overview of the biological functions of RNA editing on gene expression, diseases, cancers, and drugs. The bioinformatics resources of RNA editing were also summarized.

## 1. Introduction

RNA editing is the change of nucleotide sequence of RNA transcripts relative to that of the encoding DNA [1]. RNA editing can enhance the RNA and protein diversity [2]. Although five types of RNA editing have been discovered [3], the adenosine-to-inosine (A-to-I) editing is the most common type in higher eukaryotes [4–6]. The A-to-I editing may lead to changes in amino acid type and alternative splicing [7], thereby increasing the complexity of gene expression [8].

## 2. RNA Editing and Gene Expression

The A-to-I editing is an enzymatic process mediated by proteins of the family of adenosine deaminase acting on RNA (ADAR). Two types of A-to-I RNA editing such as site selective and hyper-editing have been summarized [9]. The site selective way usually converses one or a few A-to-I sites but the hyperediting way causes adenine deamination of long stretches of double-strand RNA [9]. Accordingly, the A-to-I RNA editing contributes a global posttranscriptional modification to the transcriptome diversity [10, 11]. A-to-I RNA editing is a common event that can lead to amino acid changes in translated exons and RNA folding or may edit in noncoding exons or introns [12, 13]. Additional gene products and functions are further generated than the original encoded genes to improve the complexity of gene expression. 

RNA editing is essential in many organisms. Correct RNA editing is important in organism's development [11]. For example, RNA editing deficiency may display the deleterious phenotypes in plant and in mammals. For example, RNA editing mutant was reported with strong defects in organelle development [14] and with pollen abortion in male sterility [15]. A RNA editing deficiency of glutamate receptor subunit GluR2 was reported in motor neurons of amyotrophic lateral sclerosis [16, 17]. The deficiency or misregulation of RNA editing may result in the development of diseases and cancers [17] as described later.

## 3. RNA Editing and Diseases

Since the RNA editing is essential in regulating gene expression of organisms, imbalance of RNA editing may lead to dysfunction of some proteins involved in normal physiology such as neural and immune functions. A large number of nervous system targets such as neurotransmitter receptors and ion channels [18, 19] undergo A-to-I RNA editing by ADARs [20]. For example, the RNA editing of serotonin (5-hydroxytryptamine (5-HT)) 2C receptor (HTR2CR) was altered in a depression animal model and antidepressants commonly reduced its RNA editing efficiency [21, 22]. Furthermore, the ADAR-mediated RNA editing in nervous system tissues may occur in both coding and noncoding transcriptomes [5, 13, 23, 24]. Particularly in the nervous system, editing in non-coding regions such as microRNA and 3′ untranslated regions (UTR) of mRNAs is more frequent than in coding regions [4, 23]. Therefore, correct and regulated RNA editing is important for marinating functional nervous system and avoiding neurological diseases [8, 25].

ADARs can regulate the innate immune system by editing the RNA transcripts of immune-related genes [26]. ADAR (ADAR1) may be involved in regulating the RNA editing and the replication of hepatitis delta virus (HDV). For example, the two forms of ADAR1 (ADAR1-S and ADAR1-L) are involved in HDV editing, where the ADAR1-S functions in unstimulated cells and ADAR1-L functions in IFN-alpha-stimulated cells [27]. RNA editing exhibits interactions between the host ADAR1, and structural motifs in the HDV RNA may play important roles in the HDV replication cycle [28].

## 4. RNA Editing and Cancers

The regulation of ADAR was found to depend on the differentiation status of pluripotent human embryonic stem cells [29], suggesting that A-to-I RNA editing is involved in human embryogenesis. Interfering the regulation of differentiation and apoptosis may promote carcinogenesis [30]. RNA regulation can modulate the expression of oncogenes or tumor suppressor genes [31]. A-to-I editing is responsible for structure change and base pairing features of the RNA molecule [32] and is involved in cell differentiation [33]. Accordingly, A-to-I RNA editing may contribute to cancer development and progression [34, 35].

For example, ADAR1 was downregulated when growth rates of HeLa-cell-derived tumors in xenograft model were inhibited [45]. ADAR1 deletion leads to regression of established chronic myelogenous leukemia in mice [46]. ADARB2 (ADAR3) mRNA was decreased in glioblastoma multiforme [34], suggesting that reduced A-to-I editing is involved in brain carcinogenesis. However, downregulation of ADARB1 (ADAR2) inhibited cellular proliferation of pediatric astrocytoma [47] and glioblastoma [48]. Additionally, RNA editing may not be involved in the carcinogenesis of urinary bladder cancer [49].

Recently, some cancer-related RNA editing targets were discovered such as antizyme inhibitor 1 (AZIN1) and glioma-associated oncogene 1 (GLI1). A-to-I RNA editing of AZIN1 is increased in hepatocellular carcinoma [50]. RNA editing of GLI1 transcription factor involved in Hedgehog signaling is decreased in basal cell carcinoma tumor [51]. Therefore, the imbalance in expression of ADAR enzymes is highly correlated with cancer development and progression [52, 53].

## 5. RNA Editing and Drugs

mRNA transcript diversity such as RNA editing has profound impact on drug discovery [2, 54]. In addition to main gene products, isoforms generated by RNA editing may provide additional drug targets that have preferential physiological effects. Accordingly, transcript diversity creates potentially new opportunities for drug design, development, and therapy [54].

RNA editing has been suggested to be a therapeutic target for CNS disorders [55]. For example, RNA editing of the 5HT2C receptor may affect cell signaling, drug response, and brain function [56]. A-to-I RNA editing can also modulate the drug response of some channels, such as Kv1.1 channel [2, 57]. Therefore, RNA editing of these receptor and channels may change their protein functions and become a target for disease therapy [57].

Recently, some drugs for inhibiting RNA editing enzymes were discovered. For example, novel inhibitors of* Trypanosoma brucei *RNA editing ligase 1 were reported to be potential therapeutic drugs [58, 59]. 

## 6. RNA Editing and Bioinformatics Resources

Although there is a high chance of finding natural products that could target the RNA editing enzymes or lead to the RNA editing of some target genes, such investigations are still rare. It is possible that certain natural products have the potential to be the inhibitors or modulators for RNA editing and may have impacts on the disease and cancer therapy. Therefore, we collected the bioinformatics resources of RNA editing (Table 1) to help the researchers of natural products to investigate the effect of natural products on RNA editing.

 In brief, dbRES [36] contains known RNA editing sites curated from the literature and GenBank. DARNED [37, 38] contains region-, gene-, and sequence-based inputs for RNA editing data retrieval from human and model organisms. miR-EdiTar [39] contains predicted miRNA binding sites that could be modified by A-to-I-editing, as well as A-to-I editing-induced miRNA binding sites. Both ExpEdit [40] and RNA-eXpress [41] are the annotation tools of RNA editing prediction for RNA-Seq data. For organellar RNA editing resources, GOBASE [42], REDIdb [43], and PREPACT 2.0 [44] contain the interface for RNA editing data of mitochondrion- and chloroplast-encoded sequences. Some bioinformatics resources of RNA editing in plants [60–63] were not included in Table 1 because they have little relationships to drug discovery. 

## 7. Conclusion

In the future, we expect that RNA editing studies related to natural products may be accumulating. We hope that this concept can inspire the scientific idea to connect the fields of RNA editing research and natural products for drug discovery in cancer therapy.

## Figures and Tables

**Table 1:** RNA editing bioinformatics resources.

Tools	Functions (Web sites)
dbRES [36]	A database for annotated RNA editing sites. (http://bioinfo.au.tsinghua.edu.cn/dbRES)

DARNED [37, 38]	A database of RNA editing in humans and model organisms with Wikipedia. (http://darned.ucc.ie)

miR-EdiTar [39]	A database of predicted A-to-I edited miRNA target sites. (http://microrna.osumc.edu/mireditar)

ExpEdit [40]	A webserver for human RNA editing in RNA-Seq experiments. (http://www.caspur.it/ExpEdit/)

RNA-eXpress [41]	An annotation tool for novel transcript features in RNA-Seq data including RNA editing and others. (http://www.rnaexpress.org)

GOBASE [42]	An organelle genome database with an interface for RNA editing data and others using multiple alignments. (http://gobase.bcm.umontreal.ca/)

REDIdb [43]	A database for organellar RNA editing sites. (http://biologia.unical.it/py_script/REDIdb)

PREPACT 2.0 [44]	Predicting RNA editing in organelle genome sequences with multiple references and curated RNA editing annotation. (http://www.prepact.de)
